# Development of heme protein based oxygen sensing indicators

**DOI:** 10.1038/s41598-018-30329-5

**Published:** 2018-08-07

**Authors:** Jiro Nomata, Toru Hisabori

**Affiliations:** 1 Laboratory for Chemistry and Life Science, Institute of Innovative Research, Tokyo Institute of Technology, Nagatsuta 4259, Midori-ku, Yokohama 226-8503 Japan; 2 Core Research for Evolutional Science and Technology (CREST), Japan Science and Technology Agency (JST), Chiyoda-Ku, Tokyo 102-0076 Japan

## Abstract

Oxygen is essential for aerobic life and is required for various oxygen-dependent biochemical reactions. In addition, oxygen plays important roles in multiple intracellular signaling pathways. Thus, to investigate oxygen homeostasis in living cells, we developed a genetically encoded oxygen sensor protein using the oxygen sensor domain of bacterial phosphodiesterase direct oxygen sensor protein (DosP), which was connected to yellow fluorescence protein (YFP) using an optimized antiparallel coiled-coil linker. The resulting ANA-Y (Anaerobic/aerobic sensing yellow fluorescence protein) was highly sensitive to oxygen and had a half saturation concentration of 18 μM. The ANA-Y reacts with dissolved oxygen within 10 s and the resulting increases in fluorescence are reversed with decreases in oxygen concentrations. This sensitivity of the ANA-Y enabled direct determinations of initial photosynthetic oxygen production by cyanobacteria. ANA-Y exhibits reversible fluorescence change of donor YFP following reversible absorbance change of acceptor DosH, and the operating mechanism of this ANA-Y could be used to develop various protein sensor probes for intracellular signaling molecules using natural sensor proteins.

## Introduction

Oxygen is required for numerous biochemical reactions. In particular, oxygen plays an essential role in adenosine triphosphate (ATP) production by acting as the terminal electron acceptor of the electron transport chain. Moreover, heme oxygenase catalyzes the first step of heme degradation by cleaving heme using oxygen^1^. Recent studies also demonstrate multiple regulatory roles of oxygen as a signaling molecule in various metabolic pathways^2^. For example, oxygen directly activates phosphodiesterase, which mediates the degradation of the second messenger molecule cyclic-di-GMP (c-di-GMP). Furthermore, under hypoxic conditions, transcription of hypoxia-inducible genes by hypoxia-inducible factor (HIF) is controlled by oxygen via hydroxylase^3,4^. Thus, changes in cellular oxygen concentrations have direct effects on constitutive metabolic processes. Hence, diverse intracellular oxygen sensing systems have been evolved to maintain cellular oxygen homeostasis.

To investigate the cellular oxygen dynamics, various methods have been developed for measuring the intracellular oxygen concentrations directly or indirectly in living cells. These include Clarke-type electrodes^5^, electron paramagnetic resonance (EPR) methods^6,7^, and optical methods using fluorescent^8,9^ or phosphorescent dyes^10–12^. Alternatively, genetically encoded type oxygen sensor probes were developed to estimate intracellular oxygen levels of bacteria or cultured mammalian cells by applying GFP^13^ or its variant and FMN-binding protein^14^. However, intracellular oxygen dynamics are not well understood until now, largely due to the absence of suitable oxygen sensors that can be used in living cells. Various natural oxygen sensing proteins have been associated with the regulation of oxygen related metabolic activities^15–18^. In particular, the direct oxygen sensor protein (DosP) reversibly alters its catalytic activity according to intracellular oxygen concentrations. Activated DosP converts cyclic di-GMP to linear di-GMP and its phosphodiesterase activity is regulated by oxygen binding to the heme-binding domain DosH^19^. DosH binds oxygen at physiological concentrations and releases oxygen under conditions of hypoxia^19–21^. DosH has been characterized using x-ray crystallography in both oxygen-binding and oxygen-free forms^22,23^. These reported properties of DosH may be suitable for the development of novel oxygen sensor protein probes. However, crystal structure analyses show that conformational changes of DosH with oxygen binding and release^22^ are insufficient for the design of a structural change-based FRET sensor protein, such as the calcium indicator Cameleon or the ATP indicator ATeam^24,25^.

Instead, DosH produces intense Soret and Qy peaks at 425 and 560 nm, respectively, in the absence of oxygen. Subsequent introduction of oxygen shifts these peaks to 414 and 580 nm, respectively, reflecting formation of the heme–oxygen complex^26^. Thus, we developed a system that amplifies absorption changes of DosH into changes in fluorescence intensity of yellow fluorescence protein (YFP) by conjugating DosH with Venus^27^, which is a YFP variant that has sufficient spectral overlap with DosH.

Herein, we present the molecular design of the DosH–Venus conjugate and the successful development of an unprecedented oxygen sensor protein. This anaerobic/aerobic sensor probe (ANA) can be used to sense micromolar changes in dissolved oxygen concentrations.

## Results

### Development of the oxygen sensing protein probe

**Figure 1:**
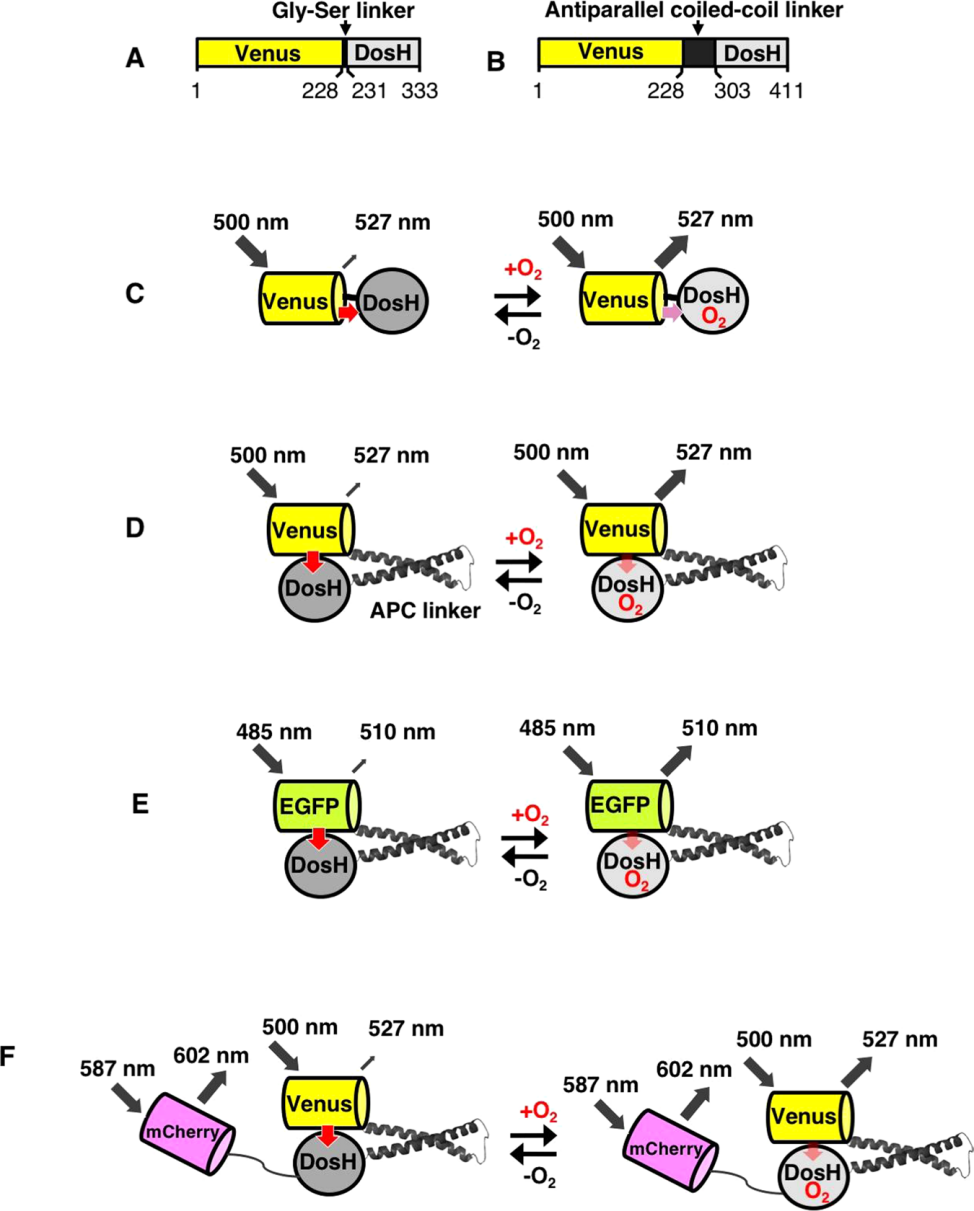
Design of the oxygen sensor protein probe. Primary structures of protoANA1 (**A**) and protoANA2 (**B**) are indicated. A variant of YFP (Venus) and the oxygen sensor domain of DosP from *E.coli* (DosH) were conjugated with a Gly-Ser linker or an antiparallel coiled-coil linker (APC linker), respectively. Fluorescence quenching mechanisms are schematically shown for protoANA1 (**C**), protoANA2 (**D**), ANA-G (**E**) and ANA-Q (**F**). In the oxygen free form, acceptor DosH exhibited a visible absorption and efficiently quenched fluorescence from donor Venus. In the oxygen-bound form, DosH had reduced absorption, leading to increased fluorescence emissions from Venus.

We designed an oxygen sensor protein by conjugating the YFP variant Venus with DosH, the DosP oxygen sensing domain from *Escherichia coli* (Fig. 1A,C). The prototype sensor protein (protoANA1) was produced by conjugating Venus and DosH using a Gly-Ser di-peptide linker. Although changes in fluorescence emission intensities at 527 nm (F527) of protoANA1 were observed following oxygen binding, the change in fluorescence signal was small (Supplementary Fig. S1A, 1.07-fold). Thus, we substituted the Gly-Ser di-peptide linker with several other linkers, including the rigid proline-rich linker^28^, bioluminescence resonance energy transfer (BRET) linker that was developed to enhance the efficiency of luciferase and YFP^29^, or the antiparallel coiled-coil linker (APC linker), which was previously used as a luciferase-based protease sensor^30^. Substitution with the APC linker (protoANA2; Fig. 1B,D) improved the rate of change slightly (Supplementary Fig. S1B, 1.11-fold).

**Figure 2:**
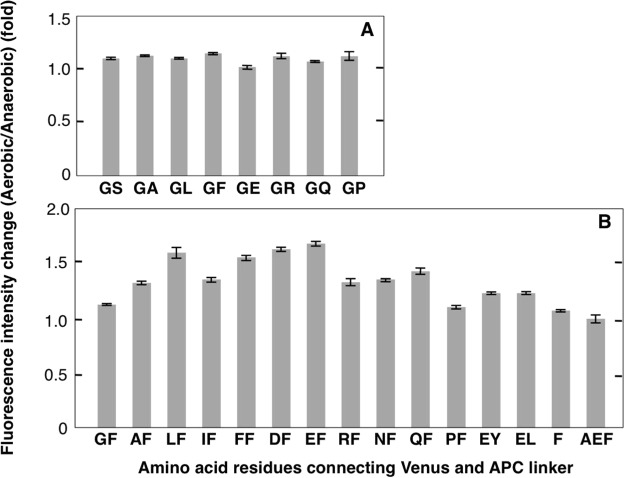
Enhancement of fluorescence intensities of ANA variants following oxygen binding. (**A**) Fluorescence intensity changes of protoANA2 with amino acid substitutions at the 230th residue (GX-variants) were measured following oxygen binding. (**B**) Fluorescence intensity changes of protoANA3 with an amino acid substitution at the 229th residue (XF-variants) were measured following oxygen binding. In (**A**) and (**B**), emission spectra of ANA variants (1.5–2 μM) were recorded in the absence or presence of oxygen, and fluorescence intensity changes (fold) were determined at 527 nm. Fluorescence intensities of ANA variants were expressed relative to that in the absence of oxygen (1.0-fold). Data are presented as means ± s.d. from three replicates.

To further improve rates of change in fluorescence signals from the oxygen sensor, we scanned the two amino acid residues (Gly^229^ and Ser^230^; Fig. 1B), which connect Venus and the APC linker. In the first scan, the Ser^230^ residue was substituted with other amino acid residues with different chemical properties, including Glu (negatively charged), Arg (positively charged), Gln (neutral), Leu (hydrophobic, bulky), Ala (hydrophobic, non-bulky), Pro (less hydrophobic), and Phe (aromatic). Protein variants were then purified and fluorescence intensity changes of the variants in the presence and absence of oxygen were determined (Fig. 2A). Fluorescence changes of the GF (Ser230Phe substitution)-variant protein were slightly improved, and this variant was designated protoANA3. In further amino acid scanning analyses, the Gly^229^ residue of protoANA3 was substituted with other residues (Fig. 2B, and the EF (Gly229Glu substitution)-variant showed the highest extent of change (1.67-fold), followed by DF-, LF- and FF-variants, which had similar extent of change of 1.65-fold, 1.59-fold, and 1.56-fold, respectively. Shorter (F^229^) or longer (A^229^E^230^F^231^) connections between Venus and the APC linker critically affected extent of change in the protoANA3 (1.07-fold to 1.01-fold, respectively), and therefore the EF-variant (Glu^229^ Phe^230^) was designated ANA-yellow (ANA-Y).

### Characterization of ANA-Y and ANA variants *in vitro*

**Figure 3:**
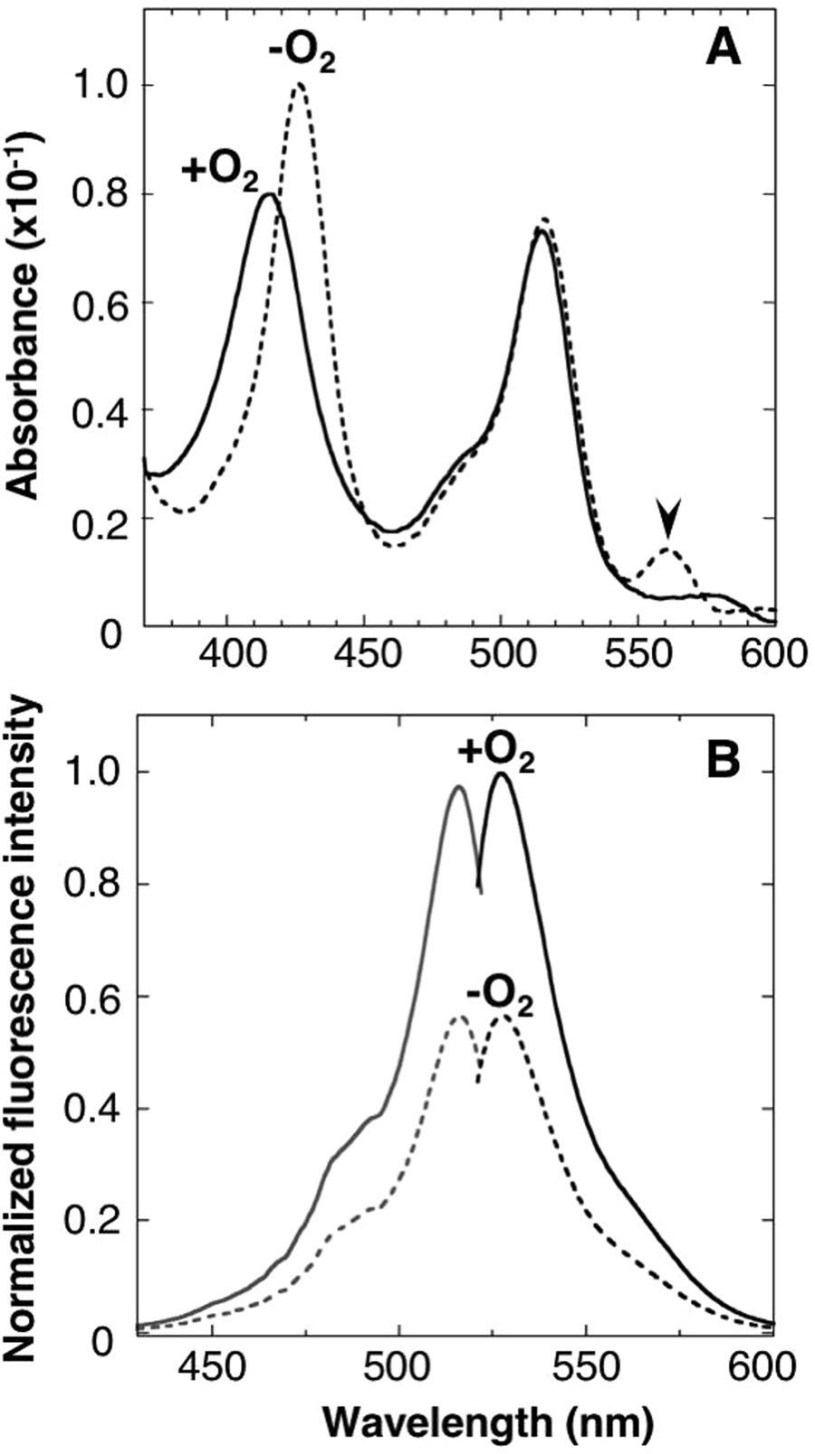
Spectroscopic properties of ANA-Y. (**A**) Absorption spectra of the ANA-Y (0.6 μM) were measured in the absence (dashed line) or presence (solid line) of 240-μM oxygen. The absorption peak of DosH at 560 nm is indicated with an arrow. (**B**) Excitation (gray color) and emission (black color) spectra of ANA-Y were measured in the absence (dashed line) or presence (solid line) of 240-μM oxygen. Excitation and emission spectra of ANA-Y (1 μM) were recorded at emission and excitation wavelengths of 527 and 500 nm,respectively. Data are expressed relative to the fluorescence intensity of ANA-Y in the presence of oxygen (1.0).

To investigate spectroscopic properties of the purified ANA-Y, absorption spectra were determined in the absence and presence of oxygen (Fig. 3A). In these analyses, intense Soret (414 or 425 nm) and Qy (560 or 580 nm) peaks are produced by heme bound to the DosH-part of the ANA-Y, and the 515-nm peak indicated Venus absorption. In the absence of oxygen, ANA-Y showed a Soret peak at 425 nm and a Qy peak at 560 nm, indicating that the bound heme in DosH-part was oxygen free^26,31^. After aeration of the solution for 10 s, shifts in Soret and Qy peaks to 414 and 580 nm indicated rapid formation of heme–oxygen complexes^26,31^. Thus, we recorded fluorescence excitation and emission spectra of ANA-Y, in the absence and presence of oxygen (Fig. 3B). Aeration of the ANA-Y solution led to a substantial increase (about 1.7-fold) in the fluorescence excitation peak at 515 and in emissions at 527 nm.

To confirm that the oxygen-dependent fluorescence changes of ANA-Y are caused by the absorption change of heme bound to DosH, we generated a heme-free variant by substituting the heme-chelating His^348^ residue of DosH with Ala (H348A variant, see Supplementary Fig. S2A). The purified H348A variant showed no absorption at 414 or 580 nm under aerobic conditions, indicating the lack of heme at the binding pocket (Supplementary Fig. S2B). Accordingly, no changes in fluorescence emission intensity of the H348A variant were observed with aeration of the solution (Supplementary Fig. S2C).

**Figure 4:**
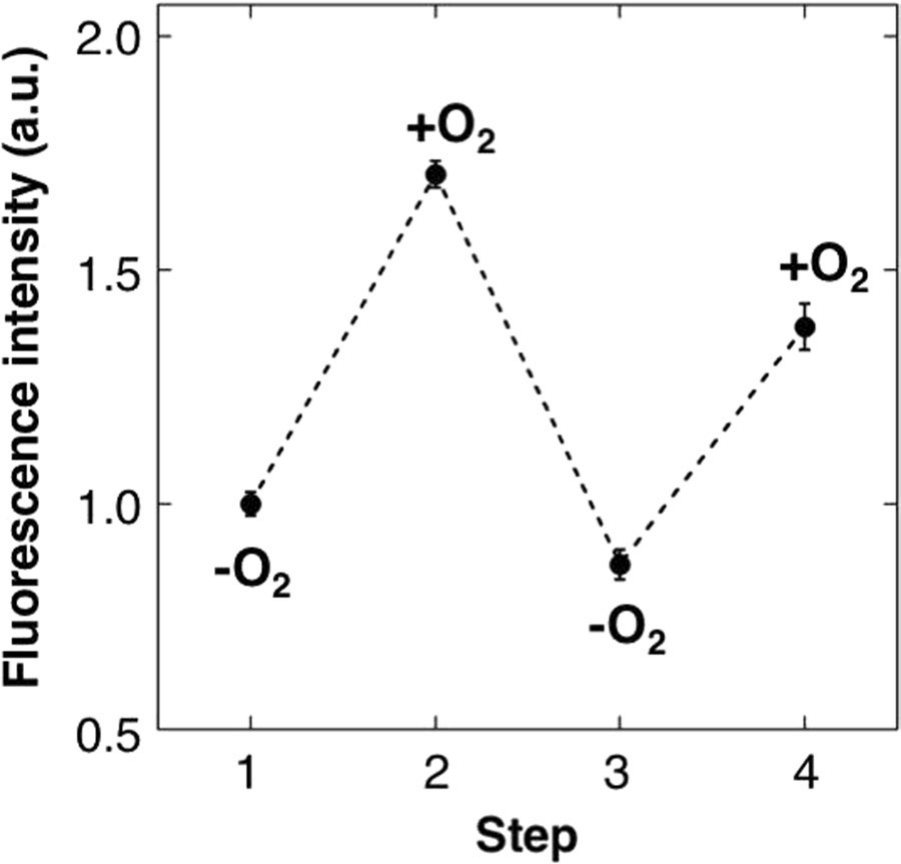
Reversibility of ANA-Y emission. Odd and even numbered steps indicate fluorescence emission intensities of ANA-Y at 527 nm in the absence and presence of 240-μM oxygen, respectively. Fluorescence intensities were normalized to protein concentrations (1 μM). Data are presented as means ± s.d. from three replicates.

In further experiments, we determined reversibility of fluorescence changes of ANA-Y against oxygen concentrations in the solution. In Fig. 4, fluorescence intensities of ANA-Y are presented relative to those under anaerobic conditions. Subsequent aeration increased fluorescence by 1.7-fold (Fig. 4 Step 2). The oxygenated ANA-Y was then transferred into anaerobic buffer by substituting the solution with Ni-NTA agarose in an anaerobic chamber. Complete removal of oxygen using sodium dithionite decreased the fluorescence of ANA-Y (Fig. 4, Step 3), and re-aeration increased fluorescence by 1.5-fold (Fig. 4, Step 4).

**Figure 5:**
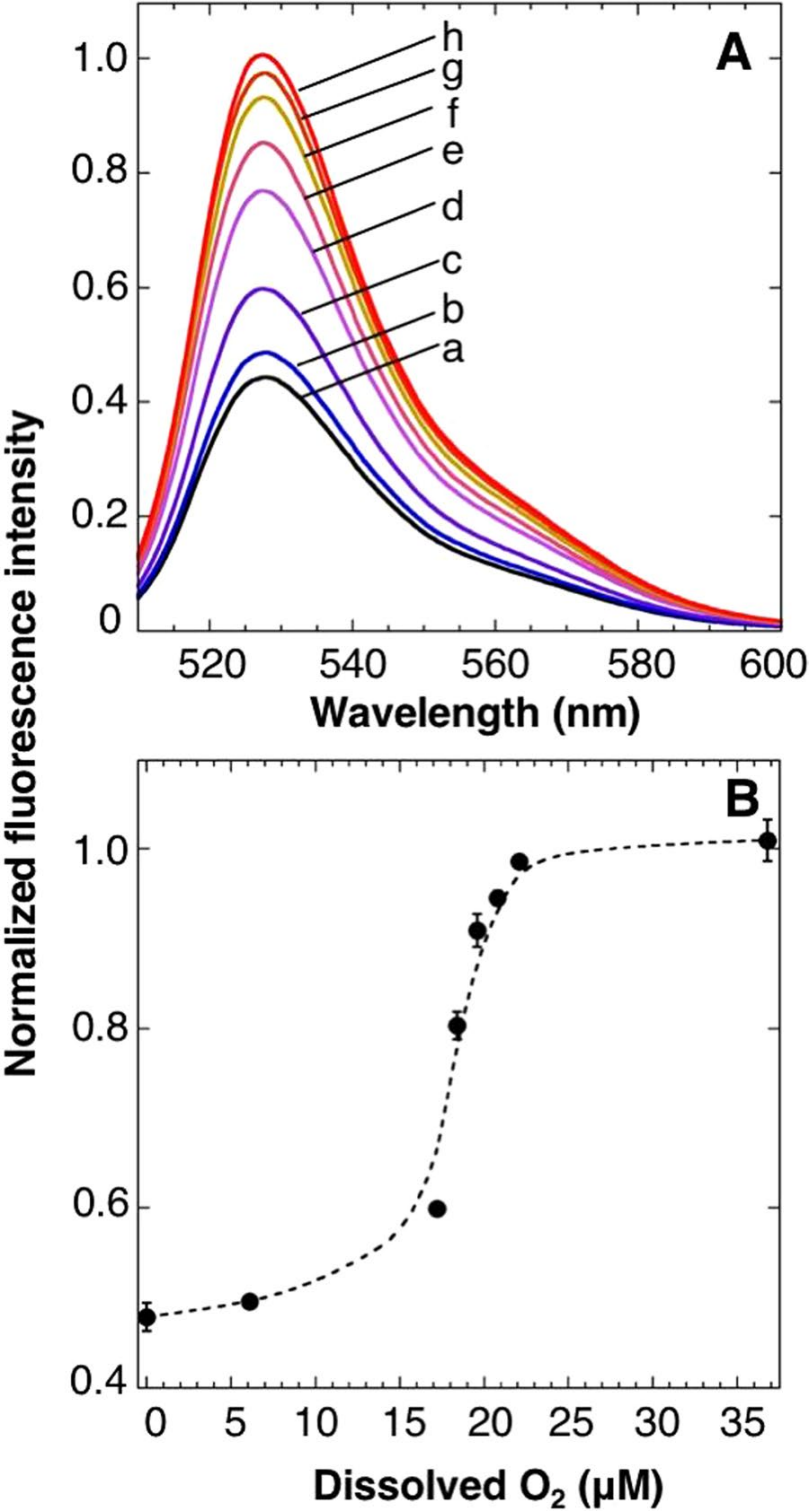
Oxygen sensitivity of ANA-Y. (**A**) Emission spectra of ANA-Y were measured in the presence of various concentrations of oxygen. Traces a, b, c, d, e, f, g, and h are emission spectra of ANA-Y in the presence of dissolved oxygen at 0, 6.1, 17.2, 18.4, 19.6, 20.8, 22.1, and 36.8 μM. (**B**) Changes in oxygen-dependent fluorescence of ANA-Y were determined from fluorescence emissions at 527 nm. Fluorescence emission intensities of ANA-Y were measured as in (**A**). Data are presented as means ± s.d. from three replicates.

To further characterize oxygen sensitivity of ANA-Y, the protein was exposed to 0–36.8 μM oxygen and fluorescence emission spectra were again analyzed (Fig. 5A). Peak values at 527 nm were then plotted against oxygen concentrations, and the half-saturation concentration of ANA-Y was determined to be 18 μM (Fig. 5B), which is comparable to *K*_*d*_ values of DosH (13–20 μM) from previous reports^20,21,26^.

To determine the fluorescence lifetime of ANA-Y, we used a fluorescence lifetime spectrometer to generate lifetime curves of ANA-Y (Supplementary Fig. S3), and then fitted these data to a double exponential decay curve. These analyses indicated lifetimes of 1.00 and 3.11 ns under anaerobic conditions, and 1.55 and 3.69 ns under aerobic conditions, respectively (Supplementary Table S1). The longer lifetimes of 3.11 and 3.69 ns correspond with those reported previously for Venus (3.25 ns^32^), suggesting that purified ANA-Y includes heme-free fraction, and it reflects the relatively lower heme-binding rates of ANA-Y (Supplementary Table S2). In addition, the shorter lifetime component was increased by 1.6-fold upon addition of oxygen (1.00 ns to 1.55 ns), reflecting the changes in fluorescence intensity shown in Fig. 3B.

**Figure 6:**
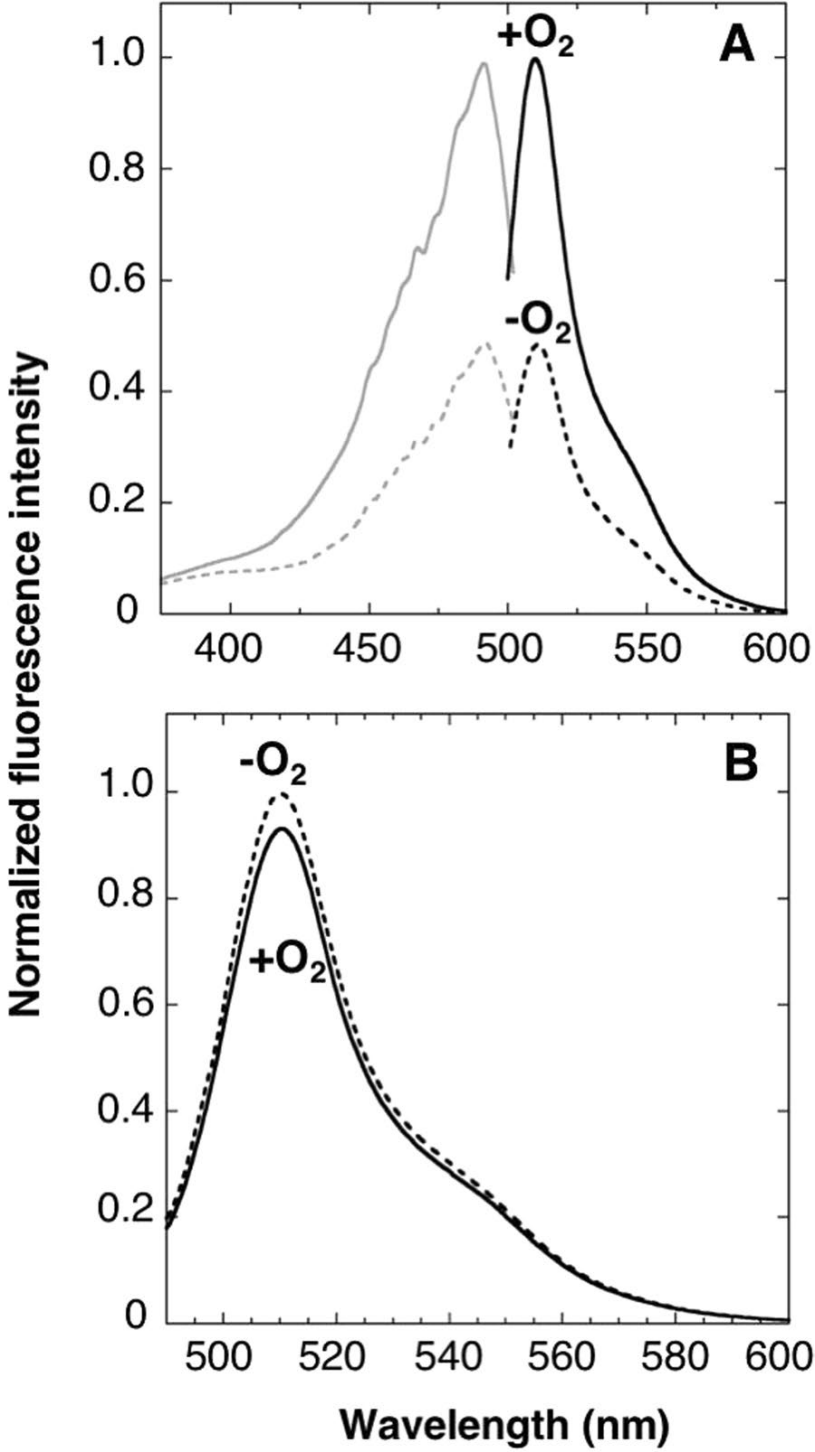
Spectroscopic properties of ANA-G. (**A**) Excitation (gray color) and emission (black color) spectra of ANA-green (ANA-G; 1 μM) were measured in the absence (dashed line) or presence (solid line) of 240-μM oxygen. Excitation and emission spectra of ANA-G were measured at emission and excitation wavelengths of 510 and 490 nm, respectively. (**B**) Emission spectra of ANA-G His348Ala variant (1 μM) in the absence (dashed line) or presence (solid line) of 240-μM oxygen; Data are presented relative to the fluorescence intensity of ANA-G in the presence of oxygen (1.0).

To expand the color diversity of the oxygen sensor protein, we conjugated enhanced green fluorescence protein (EGFP^33^) with DosH using the APC linker, producing a Glu^229^-Phe^230^ connection between EGFP and the linker, and determined fluorescence excitation and emission spectra of the resulting ANA-green (ANA-G) protein (Figs 1E and 6A). Aeration of ANA-G solution led to substantial increases (2.1-fold) in fluorescence excitation at 490 nm and emission at 510 nm, and these changes were heme-dependent because the heme deficient mutant ANA-G containing His348Ala variant DosH did not show any O_2_-dependent changes in fluorescence signal (Fig. 6B).

The pH dependence of the fluorescence intensity change of ANA-Y and ANA-G was examined (Supplementary Fig. S4). Although both sensors showed slight pH dependence, they are functional under all pH conditions examined, and both sensors showed maximum dynamic range at pH 8.0.

Relative brightness of ANA-Y or ANA-G was determined by comparing fluorescence intensity of sensor with that of Venus or EGFP, respectively. ANA-Y showed 15% or 25% of relative brightness, and ANA-G showed 12% or 24% of relative brightness, in the absence or presence of oxygen, respectively (Supplementary Fig. S5)

**Figure 7:**
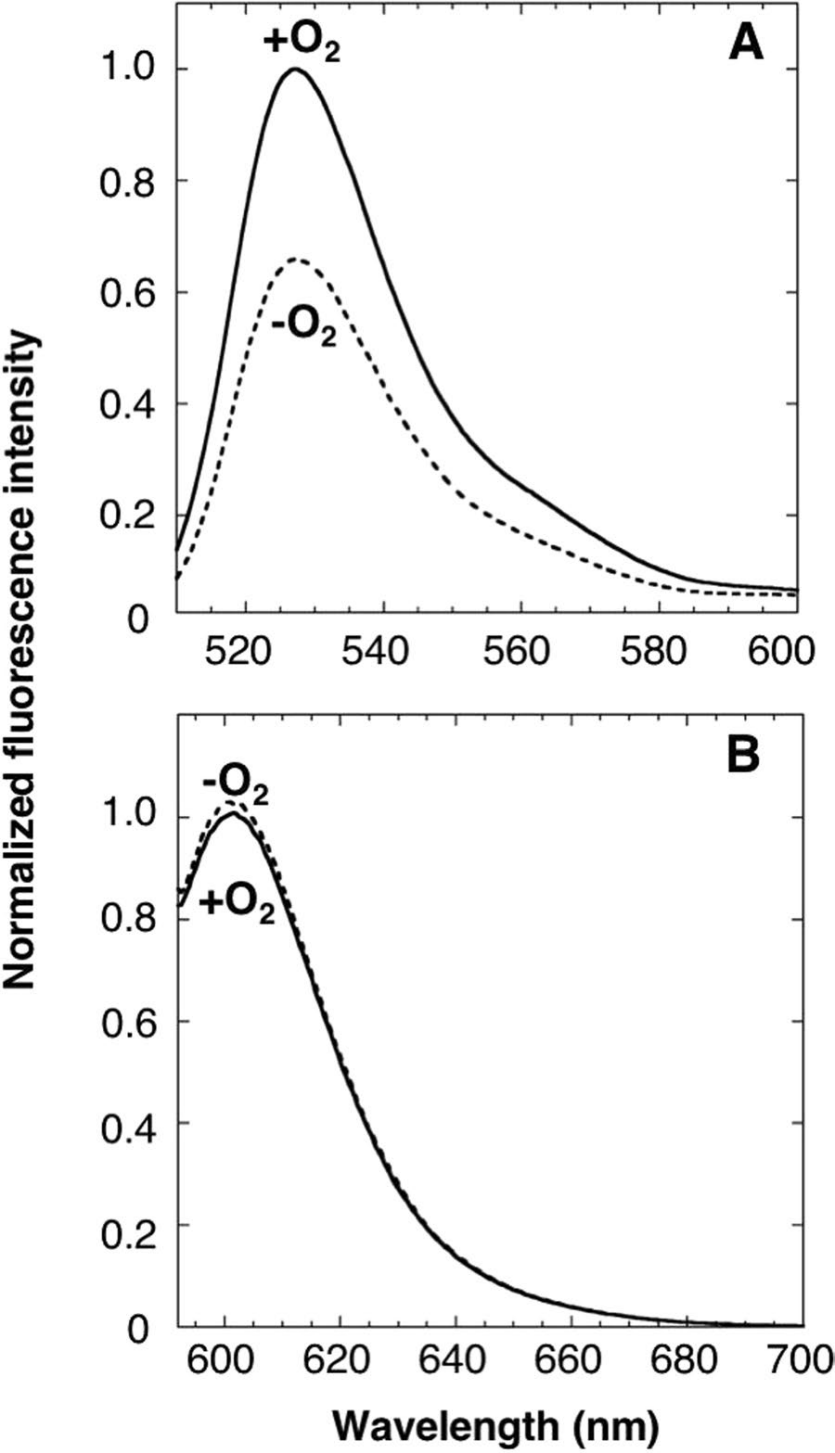
Spectroscopic properties of ANA-Q. Emission spectra of ANA-quantity (ANA-Q; 1 μM) were measured in the absence (dashed line) or presence (solid line) of 240-μM oxygen at excitation wavelengths of (**A**) 500 nm or (**B**) 587 nm, respectively. Data are presented relative to the fluorescence intensity of ANA-Q in the presence of oxygen (1.0).

To engage the quantification of dissolved oxygen in living cells, we generated the ratio metric type oxygen sensor protein by conjugating ANA-Y with a red fluorescence protein mCherry, and designated the resulting protein ANA-quantity (ANA-Q; Fig. 1F). Aeration of ANA-Q solution led to an increase (1.6-fold) in the fluorescence emission peak at 527 nm (Fig. 7A), and the emission peak of mCherry at 602 nm was not changed as expected (Fig. 7B). Consequently, the emission ratio (527/602 nm) increased by 1.6-fold upon aeration.

### Monitoring of photosynthetic oxygen production in cyanobacteria using ANA sensors

**Figure 8:**
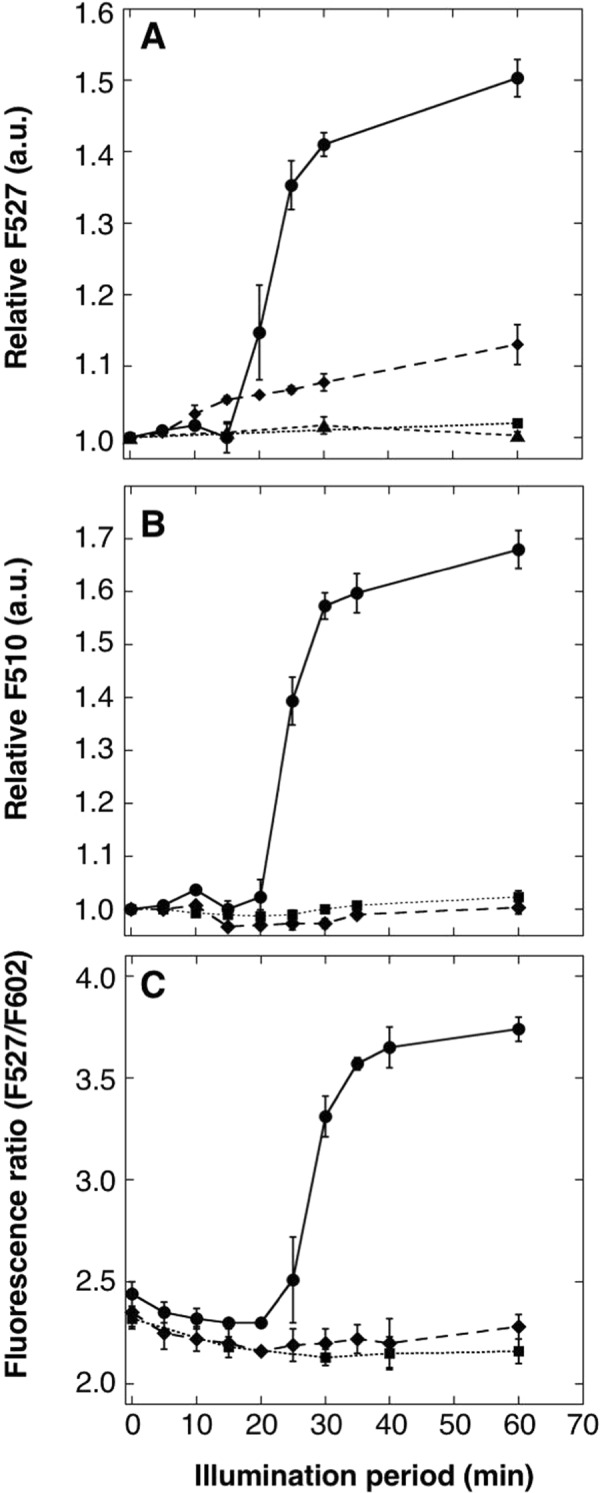
Monitoring of photosynthetic oxygen production in cyanobacteria using ANA sensors. Time courses of changes in emission intensities of (**A**) ANA-Y, (**B**) ANA-G and (**C**) ANA-Q solution suspending A.7120 cells; suspensions of A.7120 cells (1.2–2.4 μg Chl mL^−1^) in the presence (diamond) or absence (circle) of 10-μM DCMU (ANA-Y) or 12.5-μM DCMU (ANA-G or ANA-Q) were illuminated at 13 μmol photons m^−2^ s^−l^ and emission intensities of ANA-Y (1.7 μM) at 527 nm, ANA-G (1.7 μM) at 510 nm and ANA-Q (1.0 μM) at 527 nm and 602 nm were measured. Solution without A.7120 cells were used as a negative control (square). Time courses of emission intensity changes of Venus in A.7120 cells suspension was measured as a control (**A**, triangle). Fluorescence intensity data are presented relative to that of ANA-Y or ANA-G at time 0 (1.0), or fluorescence ratio of F527/F602. All data represent means ± s.d. from three replicates.

ANA-Y, ANA-G and ANA-Q were used to monitor photosynthetic oxygen evolution in cyanobacterium *Anabaena* sp. PCC 7120 (A.7120) cells. Fluorescence of ANA sensors started to increase after 20-min illumination and reached plateaus after 60 min (Fig. 8 circles). Moreover, a small increase in fluorescence intensity was observed (Fig. 8A diamonds) in the presence of the specific inhibitor of photosystem II diuron (3-(3,4-dichlorophenyl)-1,1- dimethylurea (DCMU)) at 10 μM^34^. In contrast, negligible fluorescence increases were observed in the presence of 12.5-μM DCMU (Fig. 8B,C diamonds).

## Discussions

In this study, we developed a genetically encoded oxygen sensor protein probe by combining oxygen sensor domain DosH of bacterial phosphodiesterase DosP with the YFP variant Venus. We initially fused these proteins using a short Gly-Ser linker (protoANA1), but observed only slight oxygen sensitive changes in fluorescence intensity (Supplementary Fig. S1A) and those in fluorescence lifetime (Supplementary Fig. S3, Supplementary Table S1). Thus, to increase the oxygen-sensitive fluorescence intensity change, we examined relative orientations of the donor Venus and the accepter DosH and finally connected these components using an antiparallel linker (APC linker), which is developed for a previously characterized protease sensor^30^. Fluorescence from the resulting protoANA2 had a lifetime of 1.60 ns in the presence of oxygen, indicating dramatically enhanced fluorescence quenching caused by oxygen-binding to DosH (Supplementary Fig. S3, Supplementary Table S1).

To improve the degree of change of our sensor probe, we optimized the two amino acid residues (Gly^229^ Ser^230^) connecting Venus and the APC linker using theoretical scanning techniques, and found that the use of Glu-Phe connections enhances the rate of change following oxygen binding, and the ensuing ANA-Y gave an oxygen-dependent fluorescence intensity change of 1.67-fold. (Figs 2B and 3B). 1.6-fold fluorescence lifetime change of ANA-Y following binding or release of oxygen (Supplementary Fig. S3, Supplementary Table S1) suggested that ANA-Y could be applied to fluorescence lifetime imaging analysis.

Fluorescence intensity and fluorescence lifetime change of ANA sensors following O_2_ binding indicates that ANA sensors function caused by fluorescence quenching of the heme molecule bound to DosH. It is reported that Fe^2+^ quench the fluorescence from FPs^35^ or fluorescent organic nanoparticles^36^ by the static quenching. Fe^2+^ bound to the heme molecule in ANA sensors might quench the fluorescence from Venus or EGFP in ANA-Y or ANA-G, respectively. Also, it is reported that DosH has small conformational changes upon oxygen binding and release^22^ around FG-loop. Alternatively, these changes may affect the DosH-Venus or DosH-EGFP relative orientation and efficiency in fluorescence quenching, and contribute to enhanced fluorescence intensity changes.

Importantly heme is essential for fluorescence intensity change of ANA sensors following oxygen binding. Heme binding fraction of protoANA1, protoANA2 and ANA-Y were estimated to be 79, 82 and 66% (Supplementary Table. S2), respectively, indicating that heme binding affinity of ANA-Y is relatively lower than those of protoANA1 and protoANA2. In the *E.coli* overexpression system, as purified ANA-Y exhibited impaired degree of fluorescence change (1.2-fold; Supplementary Fig. S6, free), indicating insufficient heme supply in the cells. Alternatively, supplement with a precursor for the biosynthesis of hemin, 5-aminolevulinic acid (ALA), in the culture medium enhanced the degree of fluorescence change of ANA-Y (1.63-fold; Supplementary Fig. S6, ALA), which is comparable to that of hemin-added ANA-Y (1.73-fold; Supplementary Fig. S6, hemin). These data suggested that, although supplementation of ALA in the cancerous cells or tissues potentially stimulates the accumulation of a phototoxic pigment protoporphyrin, ALA-supplementation should largely enhance the dynamic range of ANA sensors in the practical use of them.

ANA-Y exhibited 18 μM oxygen half-saturation concentration and can detect at least 6 μM oxygen (Fig. 5B). It is reported that the oxygen concentration in cortical tissue of rat or bone marrow tissue of mouse is 10–42 μM or 8–53 μM^11,12^, respectively. Also, oxygen concentration in *E.coli* cells in liquid culture is reported to be less than 80 μM^14^. These results indicate that ANA-Y should be suitable to monitor biological oxygen concentrations.

Generally, fluorescence intensity of FPs can be affected by oxygen-induced triplet state quenching. To exclude the possibility that fluorescence intensity change exhibited by ANA sensor depends on that quenching, we observed fluorescence intensity change of ANA sensors under various excitation light intensities (Supplementary Fig. S7), since the triplet state quenching is strongly dependent on excitation light intensity. The relative fluorescence intensity of ANA-Y and ANA-G or fluorescence ratio of ANA-Q remained unaffected under all excitation light conditions, indicating the effect of the oxygen-induced triplet state quenching on ANA sensors is negligible.

To examine the practical use of ANA-Y, ANA-G and ANA-Q as *in situ* oxygen sensitive indicators, we monitored photosynthetic oxygen production in A.7120 cells. These experiments showed increased fluorescence of ANA sensors from 20–60 min of illumination (Fig. 8 circles). These observed delays likely reflected the presence of vestigial dithionite in the solution, which eliminates oxygen from solution. Moreover, the photosystem II inhibitor DCMU impaired or abolished increases in signals (Fig. 8 diamonds) in cell suspensions, confirming that ANAs can be used to monitor photosynthetic oxygen production in A.7120 cells with high sensitivity.

Although ANA sensors are useful probe, chromophore maturation of ANA sensors should be considered in the practical use as same as other FPs. Potentially, fluorescence signals from ANA sensors reflect the amount of matured fluorescence protein(s). Especially in the use of ANA-Q, the ratiometric measurement of Venus and mCherry fluorescence might be affected by the time-lag of maturation of these two fluorescent proteins.

Recently, an oxygen sensor was developed by fusing FMN-binding protein with YFP to produce a fluorescent protein-based biosensor for oxygen (FluBO^14^) or a yeast fluorescent oxygen sensor (YFOS^37^). Oxygen detection mechanism of FluBO or YFOS is based on the oxygen-dependent formation of chromophore in YFP. Therefore, their fluorescence changes are slow and fatally irreversible. In contrast, ANA-Y shows reversible fluorescence change (Fig. 4) and exhibits higher oxygen sensitivity and fluorescence responses to oxygen within 10 s.

Almost all current sensor protein probes are based on conformational changes^38–41^ such as disulfide bond formation^21,42,43^ and homo- or hetero-dimerization^25,44^ following binding of substrate (effecter) or changes in microenvironments. Previously, Manioglu *et al*. measured redox states of the heme protein cytochrome c using YFP^45^. In this study, cytochrome c (Cyt_c_) was attached to YFP via a short peptide linker (Ala-Ala-Ala) and redox dependent absorption changes of heme were successfully converted to changes in fluorescence intensity of YFP. Other than Cyt_c_ or DosH, numerous natural proteins such as heme-binding globins, iron sulfur cluster–containing transcriptional factors, and flavin-binding redox sensing proteins exhibit significant absorption changes upon binding of substrate or effecter molecules, and these are associated with small or negligible structural changes. Herein, the fluorescence quenching mechanism enabled use of these proteins as signal receivers, thus expanding the possibilities for the development of novel protein sensor probes.

## Methods

### Genetic construction of protoANA1

The coding region of Venus was amplified from pET21a–Venus using polymerase chain reactions (PCR) with the primer pairs for Venus1 listed in Supplementary Table S2. The coding region of DosH was amplified from *E.coli* genomic DNA using PCR with the primer pairs for DosH1 listed in Supplementary Table S2. Restriction sites are underlined. Amplified DNA fragments were digested and cloned into NdeI and XhoI sites of pET21a (Novagen, USA) to produce recombinant proteins with C-terminal 6× histidine-tags, and DNA sequences of the resulting plasmids were confirmed using a Prism 3100 instrument (Applied Biosystems, USA).

### Genetic construction of protoANA2 and variants

The coding region of Venus was amplified from pET21a–Venus using PCR with the primer pairs for Venus2 listed in Supplementary Table S2. The coding region of DosH was amplified from *E.coli* genomic DNA using PCR with the primer pairs for DosH2 listed in Supplementary Table S3. Amplified fragments were digested and cloned into the NdeI and XhoI sites of pET21a to obtain pET21a–Venus–DosH. The coding region of the APC linker was then amplified from synthetic oligo-DNAs (Invitrogen Life Technologies) using PCR with the primer pairs for the APC linker listed in Supplementary Table S3. After digestion, amplified DNA fragments encoding the APC linker were ligated into the BamHI and EcoRI sites of pET21a–Venus–DosH to obtain pET21a–protoANA2.

ANA variant plasmids were generated using pET21a–protoANA2 as a template, and site-directed mutagenesis was performed using PCR with the primer pairs listed in Supplementary Table S3.

To generate the heme-free variants (H348A-variant), corresponding mutations were introduced into pET21a–ANA-Y using the pairs of primers listed in Supplementary Table S3. To generate the green fluorescent variant of ANA-Y, the coding region of EGFP was amplified from pET21a–EGFP using PCR with the primer pairs for EGFP listed in Supplementary Table S3. Amplified fragments were then digested and cloned into the NdeI and BamHI sites of pET21a-protoANA2 to produce the pET21a–EGFP–APC linker–DosH. The green fluorescence variant ANA-green was generated using pET21a–EGFP–APC linker–DosH as a template for site-directed mutagenesis using PCR with the primer pairs for EGFP-EF listed in Supplementary Table S3. To generate the ratio metric type sensor ANA-Q, the coding region of mCherry was amplified from pET21a–mCherry using PCR with the primer pairs for mCherry listed in Supplementary Table S3. pET21a-ANA-Y was amplified using inverse PCR with the primer pairs for pET21a-ANA-Y listed in Supplementary Table S3. Amplified mCherry-fragment was then cloned into the linearized pET21a-ANA-Y by hot fusion to produce the pET21a–ANA-Y-mCherry. DNA sequences of the all resulting plasmids were confirmed by DNA sequencing.

### Expression and Purification of protoANA1, protoANA2, protoANA3 and ANA variants

The plasmids pET21a–protoANA1, pET21a–protoANA2, pET21a-protoANA3, and pET21a–ANA variants were transformed into *E. coli* BL21 (DE3) cells and the corresponding proteins were overexpressed. Harvested cells overexpressing 6× His-tagged proteins were then suspended in solution containing 25-mM Tris-HCl (pH 8.0), 150-mM NaCl, 25-μM hemin, 1× EDTA-free protease inhibitor tablet (Roche Applied Science) and were disrupted by sonication. After centrifugation at 37,000 × g for 1 h (RP50–2 rotor), supernatants were loaded onto a Ni-NTA Sepharose column (2 ml of Ni-NTA Sepharose, QIAGEN, USA) that had been equilibrated with wash buffer containing 25-mM Tris-HCl (pH 8.0) and 150-mM NaCl. After washing in buffer containing 25-mM Tris-HCl (pH 8.0), 150-mM NaCl, and 20-mM Imidazole, 6× His-tagged protein was eluted from the column using elution buffer containing 25-mM Tris-HCl (pH 8.0), 150-mM NaCl, and 250-mM Imidazole. Imidazole was then removed using an Amicon ultra centrifugal filter unit 30 K (Merck-MilliPore, Germany). Heme content of purified ANA proteins was determined by spectrophotometric method^46^.

### Spectroscopic analysis

Absorption spectra were measured using a JASCO V-550 spectrophotometer (JASCO, Japan). Fluorescence excitation and emission spectra were recorded using a Jasco-FP8500 fluorospectrophotometer (Jasco, Japan). When indicated, neutral density (ND) filters (25% or 6%) were used to reduce the excitation light intensity. To prepare oxygen-free ANA protein solutions, purified ANA proteins (0.6–2 μM) were incubated with sodium dithionite (0.5 mM) in 50-mM Tris-HCl (pH 8.0) and 150-mM NaCl for 10 s to remove dissolved oxygen in an anaerobic chamber (Coy, Glass Lake, MI) and were then diluted 20 fold. Oxygen-free ANA solution was then added to a cuvette with an air-tight screw cap in an anaerobic chamber, and absorption and fluorescence spectra were recorded. After removing the screw cap and mixing with air for 10 s, absorption and fluorescence spectra of oxygen-bound ANA were recorded.

### Reversibility of ANA-Y

After measuring fluorescence spectra, oxygen-free ANA-Y was exposed to air for 10 s and fluorescence spectra were again recorded. Subsequently, solutions of oxygen-bound ANA-Y were transferred into anaerobic chambers and were repurified using Ni-NTA agarose to concentrate the ANA-Y protein. Sodium dithionite (0.5 mM) was then added to the eluted protein solutions to remove oxygen, and fluorescence spectra were again determined. Finally, fluorescence spectra were recorded again after exposing ANA-Y to air.

### Oxygen sensitivity of ANA-Y

Solutions of oxygen-free ANA-Y at a final concentration of 1 μM were mixed with air-saturated buffer (240-μM O_2_, 23 °C) to achieve oxygen concentrations of 6.1, 17.2, 18.4, 19.6, 20.8, 22.1, and 36.8 μM. Subsequently, solutions were placed in cuvettes in an anaerobic chamber and fluorescence spectra were recorded.

### Fluorescence lifetime measurements

Fluorescence lifetime of ANA variants (1 μM) were measured using a fluorescence lifetime spectrometer Quantaurus-Tau (Hamamatsu Photonics). In all measurements, the excitation wavelength was 470 nm and fluorescence emissions were determined at 538 nm. Fluorescence lifetimes were then calculated using a single exponential curve fitting (protoANA1 and protoANA2) or double exponential fit (ANA-Y).

### Monitoring of photosynthetic oxygen production in cyanobacteria using ANAs

*Anabaena* sp. PCC 7120 (A.7120) cells were collected by centrifugation at 1,500 × g for 5 min and were washed with 10-mM HEPES-KOH (pH 7.4). Cells were then resuspended in buffer containing 10-mM HEPES-KOH (pH 7.4), 100-mM NaCl, and oxygen-free ANAs, and the solution was placed in a cuvette in an anaerobic chamber. Finally, A.7120 cell suspensions (2.4, 1.9, 1.2 μg Chl mL^−1^ for ANA-Y, ANA-G or ANA-Q, respectively) with and without 10-μM (for ANA-Y) or 12.5-μM (for ANA-G and ANA-Q) diuron (3-(3,4-dichlorophenyl)-1,1- dimethylurea; DCMU) were illuminated at 13 μmol photons m^−2^ s^−l^ and changes in respective fluorescence intensity were monitored over time.

## Electronic supplementary material

Supplementary figures (s1-s8) and tables (s1-s3)
