# Supplementary material for: Development of heme protein based oxygen sensing indicators
Source: Sci Rep. 2018 Aug 7;8:11849. doi: 10.1038/s41598-018-30329-5 (PMC6081431; doi:10.1038/s41598-018-30329-5)
Supplement: Supplementary file 1 — Supplementary figures (s1-s8) and tables (s1-s3) [file 41598_2018_30329_MOESM1_ESM.pdf]

Development of heme protein based oxygen sensing indicators

Jiro Nomata and Toru Hisabori

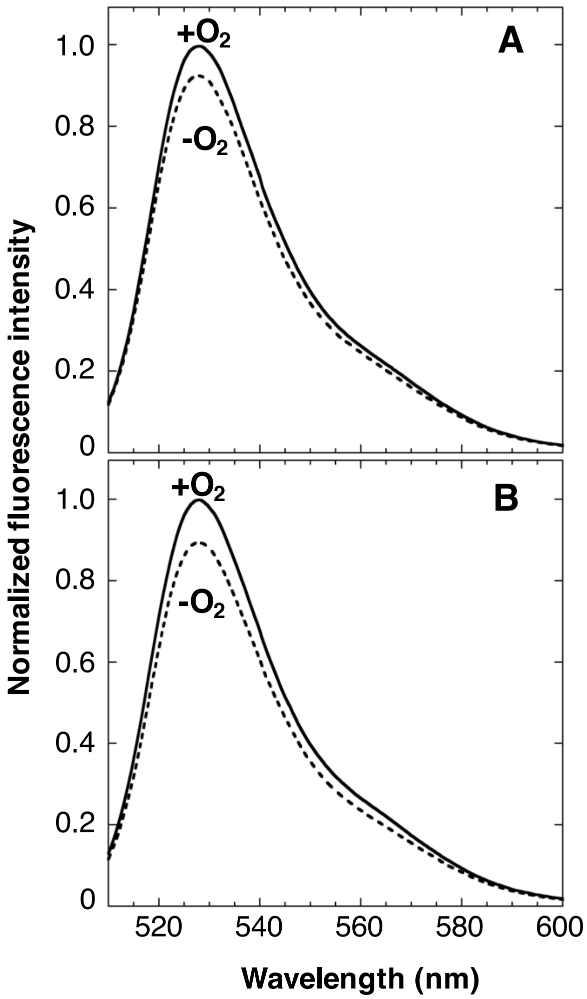

Supplementary Figure S1. Changes in fluorescence intensities of protoANA1 and protoANA2. (A) Normalized emission spectra of protoANA1 (2 µM) in the absence (dashed line) or presence (solid line) of 240 µM oxygen; (B) normalized emission spectra of protoANA2 (2 µM) in the absence (dashed line) or presence (solid line) of 240 µM oxygen; fluorescence intensity of protoANA1 or protoANA2 in the presence of oxygen was set to 1.0.

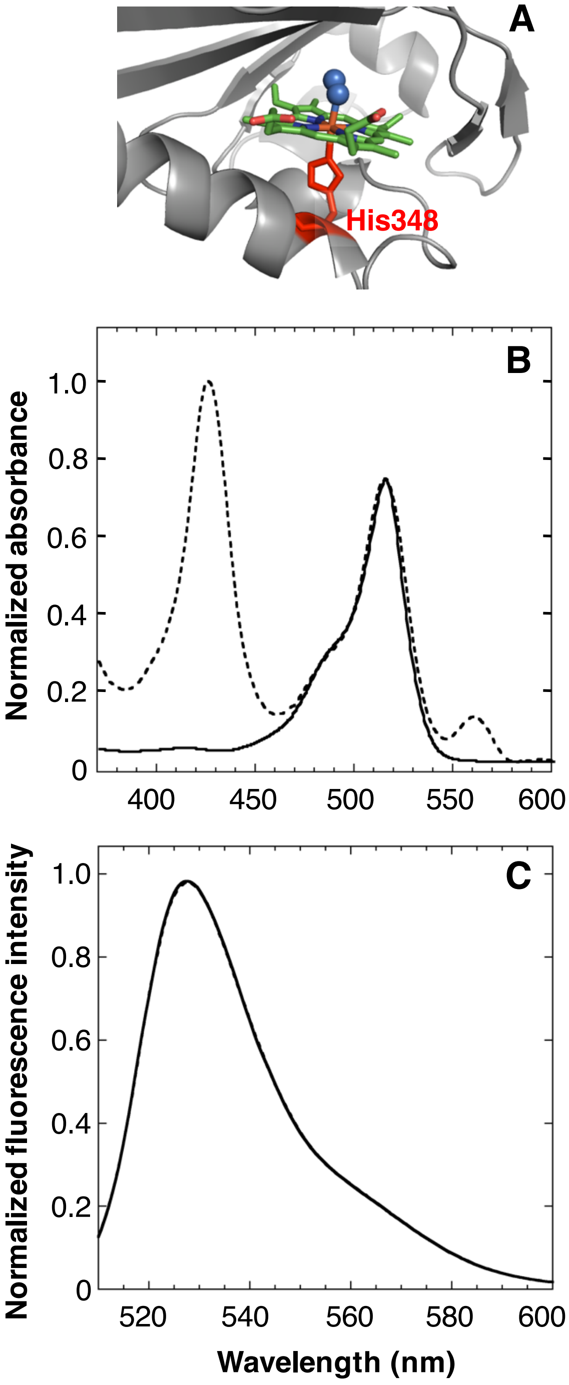

Supplementary Figure S2. Heme-dependent changes in fluorescence intensities of ANA-Y. (A) Close-up view of the DosH three-dimensional structure showing His^{348} (originally His^{77} in DosH) coordinated to the heme; Heme and His^{348} are shown as green and red sticks, respectively. Oxygen bound to heme is represented by cyan spheres. (B) Absorption spectra of the His348Ala variant (0.5 µM) were measured. The intense 515-nm peak indicates Venus absorption. Absorption spectra of ANA-Y are overlapped for comparison (dashed line). (C) Emission spectra of His348Ala variant (1 µM) in the absence (dashed line) or presence (solid line) of 240-µM oxygen were completely overlapped.

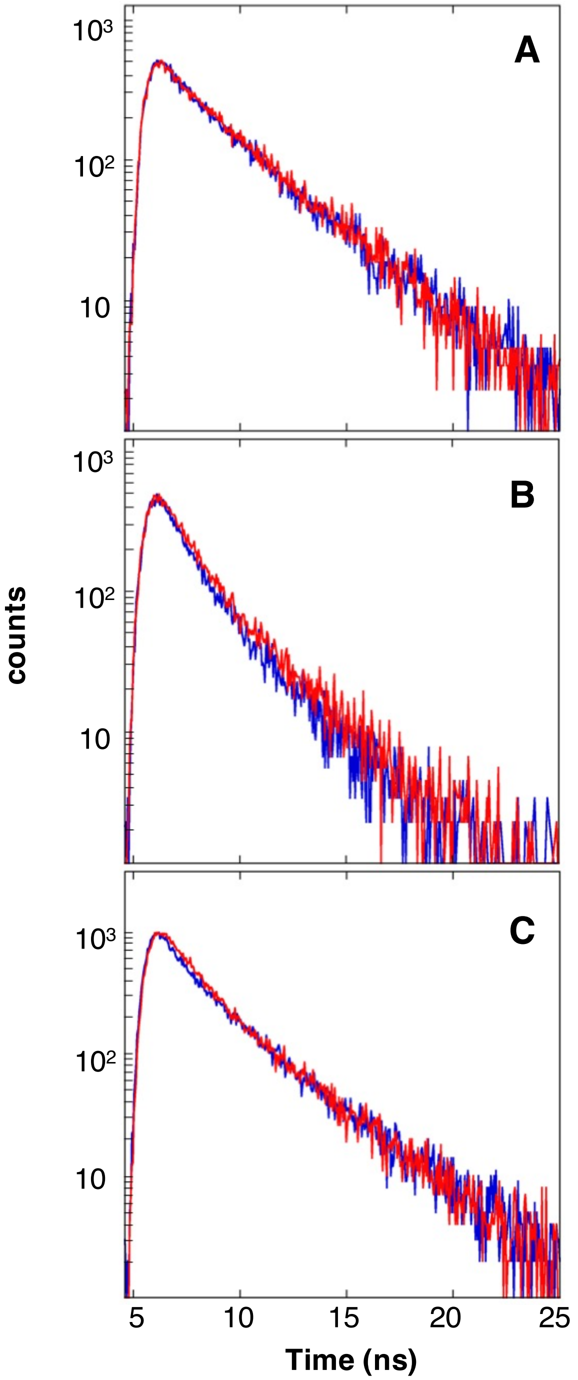

Supplementary Figure S3. Lifetime curves of protoANA1, protoANA2 and ANA-Y. Fluorescence lifetime curves of ptoroANA1(A), protoANA2 (B) or ANA-Y(C) in the absence (blue line) or presence (red line) of 240-µM oxygen.

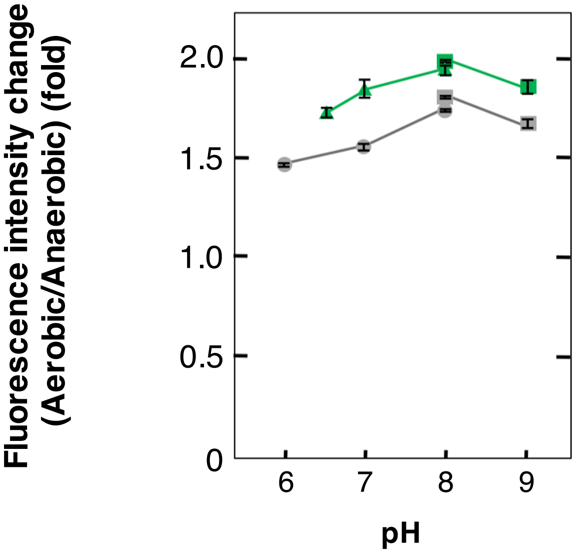

Supplementary Figure S4. pH-dependence of ANA-Y and ANA-G. Fluorescence intensity change (aerobic/anaerobic) of ANA-Y (gray) or ANA-G (green) following binding of oxygen as a function of pH. The buffer contained 50 mM MOPS-NaOH (pH 6.5, 7, 8; triangle), 50 mM sodium phosphate (pH 6, 7, 8; circle) or Tris-HCl (pH 8,9; square) and 150 mM NaCl. Data are presented as means ± s.d. from three replicates.

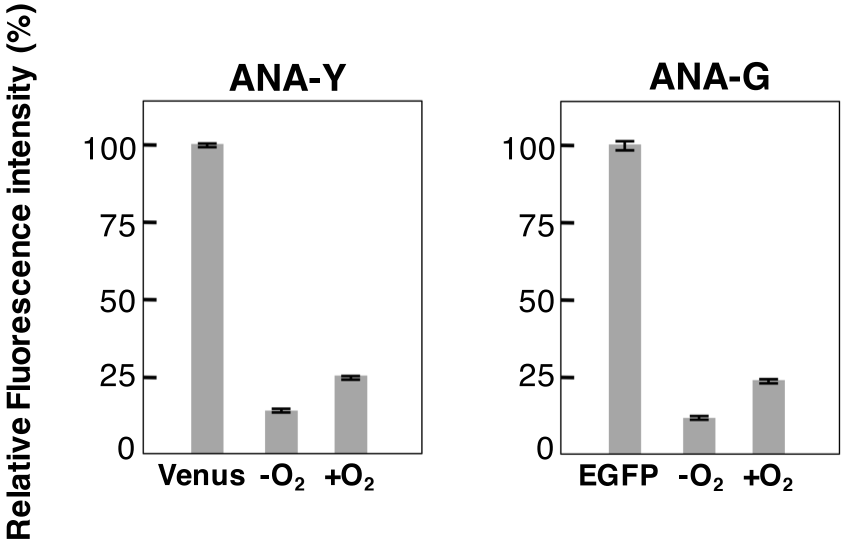

Supplementary Figure S5. Relative brightness of ANA-Y and ANA-G. Relative fluorescence intensities of ANA-Y (1 µM) or ANA-G (1 µM) in the absence (-O_2) or presence (+O_2) of 240-µM oxygen relative to Venus (1 µM) or EGFP (1 µM) in the presence of oxygen. Data are presented as means ± s.d. from three replicates.

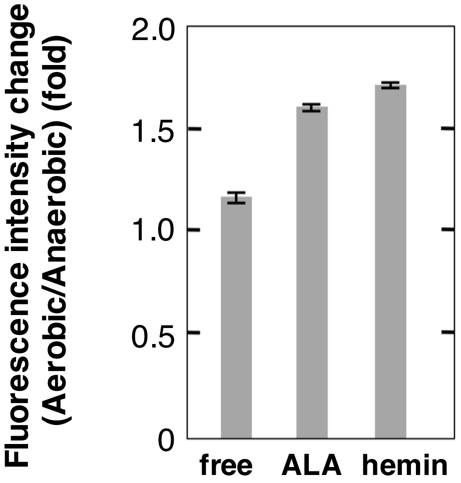

Supplementary Figure S6. Enhanced fluorescence intensity change of ANA-Y by the supplementation of 5-aminolevulinic acid in the culture medium. LB-medium supplemented with 300 μM 5-aminolevulinic acid was used to enhance the production of heme in *E.coli* cells overexpressing ANA-Y protein. Harvested cells were disrupted and ANA-Y protein was purified without addition of hemin, and fluorescence intensity change of ANA-Y (1 μM) following binding of oxygen was determined (**ALA**). For comparison, ANA-Y was overexpressed in the LB-medium without 5-aminolevulinic acid. Harvested cells were disrupted and ANA-Y was purified with or without addition of hemin, and fluorescence intensity change (aerobic/anaerobic) of ANA-Y (1 μM) was determined (**free** or **hemin**, respectively). Data are presented as means ± s.d. from three replicates.

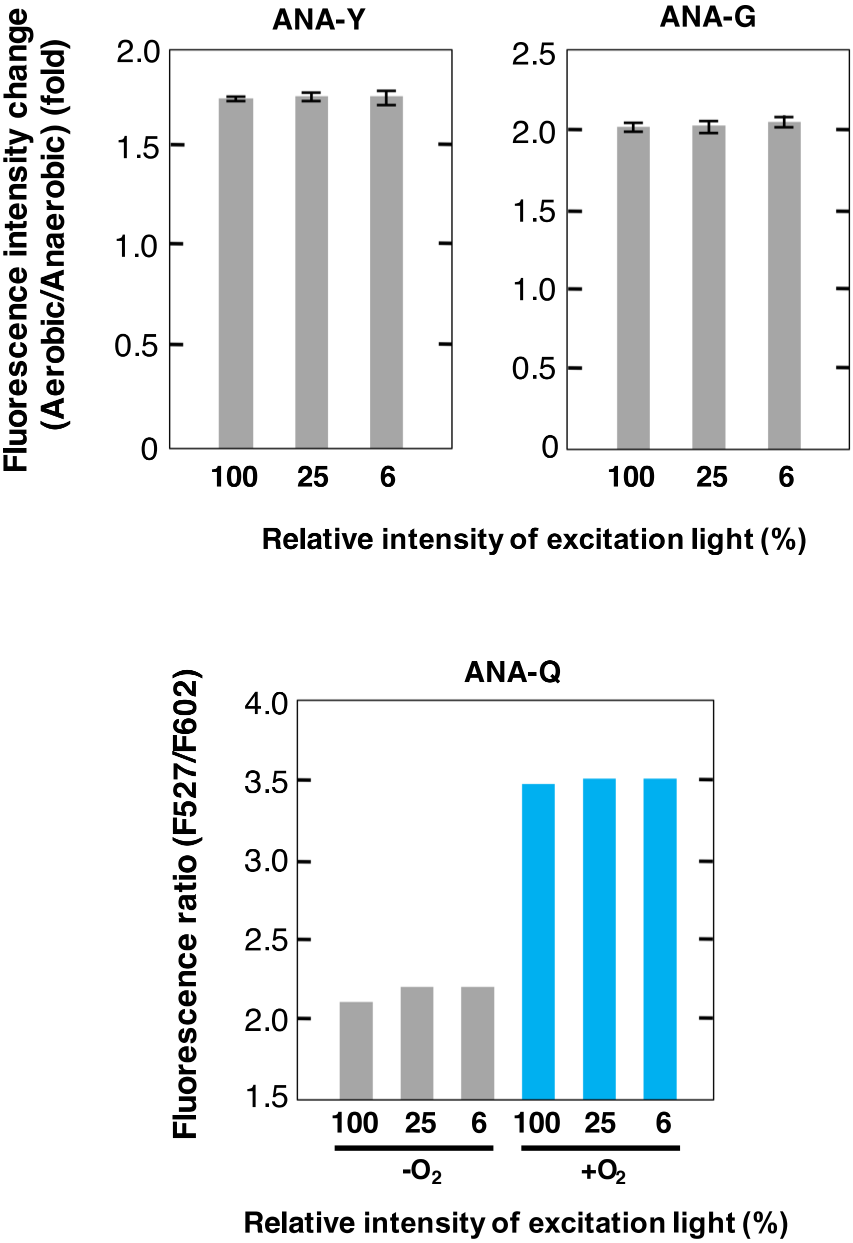

Supplementary Figure S7. Relation between the excitation light intensity and the fluorescence intensity change of ANA-Y, ANA-G and ANA-Q. Fluorescence intensity changes (aerobic/anaerobic) of ANA-Y (0.5 μM) or ANA-G (0.6 μM) were determined under the various excitation light intensities (100%, 25%, 6% of original light intensities). The original excitation light intensity was 1.6 W/cm^2 . Fluorescence ratio (F527/F602) of ANA-Q (0.5 μM) in the absence (-O_2) or presence (+O_2) of 240 mM oxygen were determined. Data are presented as means ± s.d. from three replicates (ANA-Y and ANA-G) or average of duplicates (ANA-Q).

Supplementary Table S1. Fluorescence lifetime of protoANA1, protoANA2, and ANA-Y

Oxygen sensor probes	Oxygen	Fluorescence lifetime (ns)
protoANA1	-	2.55 ± 0.02
	+	2.62 ± 0.01
protoANA2	-	1.46 ± 0.01
	+	1.60 ± 0.01
ANA-Y	-	1.00 ± 0.10 (3.11 ± 0.11)
	+	1.55 ± 0.08 (3.69 ± 0.26)
Venus	+	3.25

Supplementary Table S2. Heme binding fraction of protoANA1, protoANA2, and ANA-Y

Oxygen sensor probes	Heme binding fraction (%)
protoANA1	79
protoANA2	82
ANA-Y	66

Supplementary Table S3. Oligonucleotide primers.

name	forward primer	reverse primer
Venus1	5′-AAACATATGGTGAGCAA<br>GGGCGAGGAGCTGTTCAC-3′	5′-AAGGATCCGGCGGCGGT<br>CACGAACTCCAGCAGGAC-3′
DosH1	5′-CCGGATCCGGTGCGGTGT<br>TAATTAACGAAAATGATG-3′	5′-AAACTCGAGATCCC<br>GTACCAGCGCCAGG-3′
Venus2	5′-AAACATATGGTGAGCAA<br>GGGCGAGGAGCTGTTCAC-3′	5′-CCGAATTCGGATCCGGC<br>GGCGGTCACGAACTCCAG-3′
DosH2	5′-CCGAATTCGGTGCGGTGT<br>TAATTAACGAAAATGATG-3′	5′-AAACTCGAGATCCC<br>GTACCAGCGCCAGG-3′
APC linker	5′-CGCGCCAGTATAAGTCT<br>TCTAATGGACCGGATCC-3′	5′-TGATTAGCTACAGCTAT<br>CTTCGAATGAATTCACC-3′
EGFP	5′-TCACATATGGCTAGCA<br>AAGGAGAAGAACTCTTC-3′	5′-AAAAAGGATCCAGCAGC<br>TGTTACAAACTCAAGAAG-3′
EGFP-EF	5′-TCTTGAGTTTGTAACAG<br>CTGCTGAATTTGCACAGC-3′	5′-GCAAATTCAGCAGCTG<br>TTACAAACTCAAGAAG-3′
GA-variant	5′-TGCTGGAGTTCGTGACC<br>GCCGCCGGAGCGGCACAG-3′	5′-GTGCCGCTCCGGCGGCG<br>GTCACGAACTCCAGCAGG-3′
GL-variant	5′-TGCTGGAGTTCGTGACC<br>GCCGCCGGACTTGCACAG-3′	5′-GTGCAAGTCCGGCGGCG<br>GTCACGAACTCCAGCAGG-3′
GF-variant	5′-TGCTGGAGTTCGTGACC<br>GCCGCCGGATTCGCACAG-3′	5′-GTGCGAATCCGGCGGCG<br>GTCACGAACTCCAGCAGG-3′
GE-variant	5′-TGCTGGAGTTCGTGACC<br>GCCGCCGGAGAAGCACAG-3′	5′-GTGCTTCTCCGGCGGCG<br>GTCACGAACTCCAGCAGG-3′
GR-variant	5′-TGCTGGAGTTCGTGACC<br>GCCGCCGGACGTGCACAG-3′	5′-GTGCACGTCCGGCGGCG<br>GTCACGAACTCCAGCAGG-3′
GQ-variant	5′-TGCTGGAGTTCGTGACC<br>GCCGCCGGACAGGCACAG-3′	5′-GTGCCTGTCCGGCGGCG<br>GTCACGAACTCCAGCAGG-3′
GP-variant	5′-TGCTGGAGTTCGTGACC<br>GCCGCCGGACCAGCACAG-3′	5′-GTGCTGGTCCGGCGGCG<br>GTCACGAACTCCAGCAGG-3′
AF-variant	5′-GCTGGAGTTCGTGACCG<br>CCGCCGCATTTGCACAGC-3′	5′-GCAAATGCGGCGGCGGT<br>CACGAACTCCAGCAGGAC-3′
LF-variant	5′-GCTGGAGTTCGTGACCG<br>CCGCCCTCTTTGCACAGC-3′	5′-GCAAAGAGGGCGGCGGT<br>CACGAACTCCAGCAGGAC-3′
IF-variant	5′-GCTGGAGTTCGTGACCG<br>CCGCCATCTTTGCACAGC-3′	5′-GCAAAGATGGCGGCGGT<br>CACGAACTCCAGCAGGAC-3′
FF-variant	5′-GCTGGAGTTCGTGACCG<br>CCGCCTTCTTTGCACAGC-3′	5′-GCAAAGAAGGCGGCGGT<br>CACGAACTCCAGCAGGAC-3′
DF-variant	5′-GCTGGAGTTCGTGACCG<br>CCGCCGATTTCGCACAGC-3′	5′-GCGAAATCGGCGGCGGT<br>CACGAACTCCAGCAGGAC-3′
EF-variant	5′-GCTGGAGTTCGTGACCG<br>CCGCCGAATTTGCACAGC-3′	5′-GCAAATTCGGCGGCGGT<br>CACGAACTCCAGCAGGAC-3′
RF-variant	5′-GCTGGAGTTCGTGACCG<br>CCGCCCGTTTTGCACAGC-3′	5′-GCAAAACGGGCGGCGGT<br>CACGAACTCCAGCAGGAC-3′
NF-variant	5′-GCTGGAGTTCGTGACCG<br>CCGCCAACTTTGCACAGC-3′	5′-GCAAAGTTGGCGGCGGT<br>CACGAACTCCAGCAGGAC-3′

name	forward primer	reverse primer
QF-variant	5′-GCTGGAGTTCGTGACCG CCGCCCAATTCGCACAGC-3′	5′-GCGAATTGGGCGGCGGT CACGAACTCCAGCAGGAC-3′
PF-variant	5′-GCTGGAGTTCGTGACCG CCGCCCCATTTGCACAGC-3′	5′-GCAAATGGGGCGGCGGT CACGAACTCCAGCAGGAC-3′
EY-variant	5′-GCTGGAGTTCGTGACCG CCGCCGAATATGCACAGC-3′	5′-GCATATTCGGCGGCGGT CACGAACTCCAGCAGGAC-3′
EL-variant	5′-GCTGGAGTTCGTGACCG CCGCCGAACTTGCACAGC-3′	5′-GCAAGTTCGGCGGCGGT CACGAACTCCAGCAGGAC-3′
F-variant	5′-GCTGGAGTTCGTGAC CGCCGCCTTCGCACAGC-3′	5′-GCGAAGGCGGCGGTCA CGAACTCCAGCAGGAC-3′
AEF-variant	5′-TGGAGTTCGTGACCGCC GCCGCAGAATTTGCACAG-3′	5′-CAAATTCTGAGGCGGCG GTCACGAACTCCAGCAGG-3′
H348A-variant	5′-GTCCTGCGGCACCTGA ATACATTCGTCACAACCG-3′	5′-TCAGGTGCCGCAGGACG CAAATCCCGCGGAATCAG-3′
mCherry	5′-GATCTGAGGAAGAGGGT GTGAGCAAGGGCGAGGAG-3′	5′-GGTGGTGCTCGAGGCCG CCGGTGGAGTGGCGGCCC-3′
pET21a-ANA-Y	5′-CGGCCTCGAGCACCACC ACCACCACCACTGAGATC-3′	5′-CTCTTCCTCAGATCCGTGG TGGTGGTGGTGGTGTTCAAG-3′
